# Composite functional movement screen score predicts injuries in youth volleyball players: a prospective cohort study

**DOI:** 10.1038/s41598-022-24508-8

**Published:** 2022-11-23

**Authors:** Mostafa Zarei, Shabnam Soltanirad, Abdolreza Kazemi, Barbara J. Hoogenboom, Mahdi Hosseinzadeh

**Affiliations:** 1 Sport Rehabilitation and Health Department, Faculty of Sports Sciences and Health, Shahid Beheshti University, Tehran, Iran; 2 Department of Sport Injuries and Corrective Exercise, Islamic Azad University Tehran Science and Research Branch (Oloom Tahghighat), Tehran, Iran; 3 Department of Physical Education, Faculty of Literature and Humanities, Vali-E-Asr University of Rafsanjan, Rafsanjan, Iran; 4 Grand Valley State University, Grand Rapids, MI USA; 5 Department of Sport Injuries and Corrective Exercises, Sport Sciences Research Institute, No. 3, 5th Alley, Miremad Street, Motahhari Street, PO Box 1587958711, Tehran, Iran

**Keywords:** Health care, Risk factors

## Abstract

We aimed to investigate whether composite Functional Movement Screen (FMS) test scores can predict musculoskeletal injuries (MSI) in youth volleyball players. 131 national young volleyball players (Males: n = 100, age = 16.5 years, height = 1.787 m, mass = 68.32 kg; Females: n = 31, age = 13.83 years, height = 1.684 m, mass = 65.12 kg) participated in this prospective cohort study. The FMS screen was performed before starting the season. MSI and exposure data were collected during the season via each team’s certified athletic trainer. The mean FMS score and standard deviation for all volleyball players was 15.85 ± 3.31. A score of ≤ 14 was positive to predict MSI with specificity of 0.60 and sensitivity of 0.93. The odds ratio for (≤ 14/˃14) was 0.048. The relative risk for being injured was 3.46. Positive likelihood ratio was 2.34, and negative likelihood ratio was 0.11. The findings of this study demonstrated that an FMS score of ≤ 14 is an identifiable risk factor for injury in young volleyball players. The FMS can be used as a pre-season screening test to identify volleyball players who may be predisposed to sustaining MSI during the season ahead.

## Introduction

Volleyball is one of the most popular sports in the world. It is played by approximately 200 million players worldwide. Volleyball is reported as the eighth-most injury-prone sport in the age group 14 to 20 years^1^. Furthermore, as participation in this popular sport increases, the total absolute number of volleyball-related musculoskeletal injuries (MSI), healthcare costs, and the training time-loss due to injury among volleyball players is likely to increase as well. Based on the model introduced by van Mechelen, in order to design any effective prevention program, injury pattern characteristics of the sport must be understood so that the extrinsic and intrinsic risk factors related to any individual sport may be identified^2^. Excluding extrinsic risk factors like slippery surfaces and poor lighting which wield their influence from the outside^3^, intrinsic risk factors such as unstable joints, muscle weakness, and unreliable postural reflexes are related to the unique characteristics of an individual athlete that could expose them to the risk of sustaining injury^4^. Scientists in the area of sports medicine are highly interested in assessing the relationship between sports injuries and intrinsic risk factors^3^. Dynamic postural control, isometric muscle strength, muscle endurance, neuromuscular control and proprioception, joint range of motion, and various postural contributors such as navicular drop and functional movement ability have been suggested as different intrinsic risk factors^5–7^ in several sports^8–11^. The majority of injuries occur in the match environment, with rates typically increasing as the playing level increases^12^. However, professional level injury rates were reportedly less than those seen in semiprofessional participants^12^. A recent systematic review of the literature revealed acute ankle sprains to be the most common injury suffered by volleyball athletes, followed by overuse conditions of the knee, shoulder, and lower back^13^. The existing data indicate that the result of the Functional Movement Screen (FMS) test are related to the likelihood of subsequent injury in professional athletes. Therefore, exercises increasing test scores of the FMS may be useful at various stages of sports activity^14^. Compensatory fundamental movement patterns can increase the risk of injury in female collegiate athletes, and can be identified by using this functional movement screening tool^15^. The FMS is a screening tool designed for assessing fundamental movement patterns^16^. Integrating the principles of proprioceptive neuromuscular facilitation (PNF), muscle synergy, and motor learning, the FMS has been introduced as a pre-participation screening and performance measure that estimates an individual’s dynamic and functional capacity^17^. Cook et al. have also suggested the FMS can be used as a screening tool that may offer a different approach to injury prevention and performance predictability in athletic and active population groups^17,18^. The FMS, consisting of seven fundamental movement patterns (screening tests), is comprehensively explained elsewhere^17,18^.

Asymmetrical function or limited performance of fundamental movement patterns on the individual screens performed in the FMS have been hypothesized to expose athletes to the potential for injury during the participation, when compared to athletes without asymmetry or movement deviation^10,19^. Growing attention is being paid to the use of the FMS due to the presumption that movement-related dysfunctions are considered as intrinsic risk factors that can be modified by injury prevention strategies. The results of some studies suggest a negative relationship between perceived fatigue level and performance on the deep squat, hurdle line, in-line lunge, and trunk stability push-up subtest scores and in the total FMS score. Therefore, the authors suggest that screening tests such as the FMS should be employed following a match when players present with fatigue^20^. Identifying movement deviations in FMS testing can therefore be critical not only in recognizing an individual’s risk for injury but also in designing intervention programs for that individual^19^.

Currently, inconsistent evidence exists regarding whether the pre-participation or fundamental movement pattern screening test known as FMS has the ability to predict injury. Some argue that the FMS is not a predictive indicator of athletic performance. However, the DS and in-line lunge components of the FMS might be an indicator of athletic performance for certain athletic performance measures^21^. This information would specifically allow professional juvenile (high school) and young (collegiate) age volleyball players in a national premier league setting to be made aware of their potential for sustaining injury during an upcoming season. If potential risks for injury are known, athletes could try to improve their functional movement ability/performance in order to prevent injury and/or re-injury. Most of the studies investigating the volleyball injury predicting ability of FMS have been performed in the collegiate athletic setting (NCAA Division II) in which volleyball athletes were investigated among athletes of various other sports such as football, softball, basketball, soccer, baseball, softball, tennis, track and field^22^, and rowing^19^. Based on the different sport specific skills, characteristics, and rules, it is likely that types, frequencies, and mechanisms of MSI differ between athletes of various sports. It is therefore, unknown whether the findings of previous studies with varied collegiate athletes would differ from findings if a specific group of athletes were involved. The aim of the current study was to investigate whether FMS composite scores can predict MSI in youth volleyball players in a national premier league.

## Materials and methods

### Study design and participants

In this prospective cohort study, the dependent variable was group (injured, non-injured), and the independent variable was composite FMS score. A total of 131 volleyball players (Males: n = 100, age = 16.5 years, height = 1.787 m, mass = 68.32 kg; Females: n = 31, age = 13.83 years, height = 1.684 m, mass = 65.12 kg) underwent FMS testing before the 2019 season of the young national premier league. This study was carried out in accordance with The Code of Ethics of the World Medical Association (Declaration of Helsinki), and was also approved by the institutional review board of sport sciences research institute of Iran (IR.SSRC.REC.1399.071). All participants were members of one of the volleyball teams/clubs in Zanjan province for the entire competitive season and had 1.5 to 2.5 years of volleyball experience. They were all 13–20 years old. An interview on medical records was conducted by a team physician for all the participants and it was determined that none of the participants had experienced MSI within the prior 6 months nor had they experienced signs or symptoms of a concussion or post-concussion syndrome. All participants regularly trained three sessions of 2 h per week.

Players who had undergone surgery within the year prior to the study, and those who missed more than three training sessions per month were excluded. None of the participants had prior experience with the FMS. The aim, the potential risk, the benefits, and the procedure of the project were all explained with details to the players before enrolling them for participation in the study. Written informed consent was signed and obtained from all the participants and their teams/clubs coaches and coordinators. Parents of those participants under the age of 18 years old were also briefed about all the procedures of the project and their written informed consent was collected. The players were informed about their right to withdraw from participating in the study without any punishment at any time. A single rater (SSR) with one year of experience who was certified in administration of the FMS screen recorded the demographic data (age, height, weight, limb length) and the data regarding the injury history. Instructions were given following the standard administration of the FMS, players were screened, and the results were recorded. The seven FMS screens included the deep squat, in-line lunge, hurdle step, shoulder mobility, active straight-leg raise, trunk stability push-up, and rotary stability. The protocol for FMS assessment is fully described by Cook et al.^17,18^ Based on the FMS criteria, a score of 0 to 3 was assigned for each of the seven fundamental movement pattern tests. A composite score out of a total of 21 was recorded for each of the participants. Five out of the seven individual FMS tests were scored separately for the right and left sides of the body. In this case, the lowest score among the right and left sides was recorded as the final score for each individual test. The history of previous injuries as well as the history of genetic diseases of the players and their mental condition in the last 6 months were among the items included in the information registration questionnaire.

### Musculoskeletal injury and exposure recordings

The data regarding the MSI were recorded by the certified athletic trainers of the teams/clubs. MSI, and the exposure time for an approximate period of 6 months during the young national premier league in the year 2019 was recorded. An MSI was operationally defined as a condition that the players were unable to participate for at least one day in training or the competition. Only MSI that occurred during an organized practice or competition were included. Exposure is defined as the organized team practice or competition in which athletes will be participating^11^. Within this study, this was limited to volleyball-sanctioned practices and competitions occurring in the 6 month follow up period of the study. The training hours players spent practicing for or competing in an organized match were recorded.

### Statistical analysis

All data regarding the FMS test, injury, and exposure time were recorded on an Excel spreadsheet (version 14; MS Corp, Santa Rosa, California) and analyzed using SPSS (version 22; IBM Corp, Armonk, NY). Descriptive statistics were calculated for demographic data, composite FMS score, injury, and exposure data. The discrete and continues variables were converted into categorical variables. A logistic regression was carried out to assess the effect of gender, and composite FMS scores on the likelihood of sustaining injury in volleyball players of this study. To compare the results of this study with others who have considered the composite score of 14 as the cut-point of the FMS, dichotomized composite FMS scores (≤ 14 vs. ˃14) were used. Chi-square statistics were used to determine the association between injury risk and dichotomized FMS composite score (≤ 14 vs. ˃14).

Receiver Operating Characteristic (ROC) curve, Area Under the Curve (AUC), and Cross Tabulations were used to examine sensitivity (SN), specificity (SP), optimal cut-point composite score, positive likelihood ratio (LR+), negative likelihood ratio (LR−), positive predictive value (PV+), negative predictive value (PV−), odds ratios (ORs) and relative risk (RR). Additionally, a chi-square statistic was used to determine the association between injury risk & FMS composite score based on the optimal cut-point composite score of the current study. An Independent t-test was used to investigate the differences between mean values for the males and females.

### Ethical approval

This study was carried out in accordance with The Code of Ethics of the World Medical Association (Declaration of Helsinki), and was approved by the institutional review board of sport sciences research institute of Iran (IR.SSRC.REC.1399.071).

## Results

Volleyball players from four teams voluntarily participated in the study. A total of 131 participants were included (100 male and 31 female), age range 13–20 years old, mean age was 15.87 ± 1.81. One hundred four participants (79.4%) were right-footed, and 119 (90.8%) were right-handed. The mean FMS composite score for all the participants was 15.85 ± 3.31. The demographic data and baseline characteristics related to the participants are shown in Table 1. The total exposure time (volleyball-sanctioned practices and competitions) of 11,376 h occurring in the 6 month competitive season of the young national premier league in 2019 was recorded for all athletes combined. From this total exposure time, 8856 h were recorded during the exercise time, and 2520 h were during the match time. Fifty-eight players (44.3%), 46 male and 12 female volleyball players, sustained a total of 64 volleyball injuries during the six and a half month period of the league. Six participants sustained a second injury after the first one (these data were discarded during the data analysis). None of the injuries sustained outside of participation to Volleyball. Overuse and traumatic MSI represented 31% (n = 18) and 69% (n = 40), respectively, of the total MSI. Lower extremity MSI were the most frequent type in this group. An injury summary by body region and frequency is shown in Table 2.

**Table 1:** Demographic and baseline characteristics of participants.

Variable	Participants (N = 131)		
	All	Injured	Uninjured
**Gender**			
Men	100	46	54
Women	31	12	19
Age (years)	15.87 ± 1.81	15.96 ± 1.82	15.80 ± 1.82
Height (cm)	176.30 ± 7.07	176.27 ± 6.40	176.32 ± 7.61
Weight (kg)	67.56 ± 6.18	67.97 ± 5.91	67.24 ± 6.41
Hand length (cm)	88.20 ± 4.84	88.32 ± 4.43	88.11 ± 5.17
Leg length (cm)	89.79 ± 4.87	89.79 ± 4.72	89.79 ± 5.01
FMS composite score	15.85 ± 3.31	13.94 ± 3.51*	17.36 ± 2.18
Deep squat	2.46 ± 0.78	2.20 ± 0.89*	2.67 ± 0.62
Hurdle step	2.39 ± 0.67	2.32 ± 0.68	2.45 ± 0.66
In line lunge	2.15 ± 0.94	1.81 ± 1.05*	2.42 ± 0.74
Shoulder mobility	2.35 ± 0.92	2.00 ± 1.09*	2.63 ± 0.65
Active straight leg raise	2.05 ± 0.74	1.72 ± 0.72*	2.31 ± 0.66
Trunk stability push-up	2.48 ± 0.72	2.22 ± 0.83*	2.69 ± 0.54
Rotatory stability	1.94 ± 0.74	1.65 ± 0.82*	2.17 ± 0.58

*The mean differences are significant comparing to the un-injured participants at the *p* < 0.05 level.

**Table 2:** Summary of injuries by body region.

Body region	No. (%)
Lower extremity	24 (41.4%)
Trunk	20 (34.5%)
Upper extremity	12 (20.7%)
Head	2 (3.4%)
Total	58 (100%)

### Logistic regression analysis

The logistic regression carried out to assess the effect of gender, and composite FMS scores on the likelihood of sustaining injury in volleyball players of this study provided the model (X^2^ (2) = 38.609, *p* < 0.001). The overall model was statistically significant compared to the null model, and it explained 34% of the variation of getting injured (Nagelkerke R^2^; suggesting that predictions are fairly reliable) and correctly predicted 74% of injured volleyball players. Composite score (*p* < 0.001) was significant but gender (*p* = 0.91) was not.

### Receiver operating characteristics curve and area under the curve analyses

**Figure 1:**
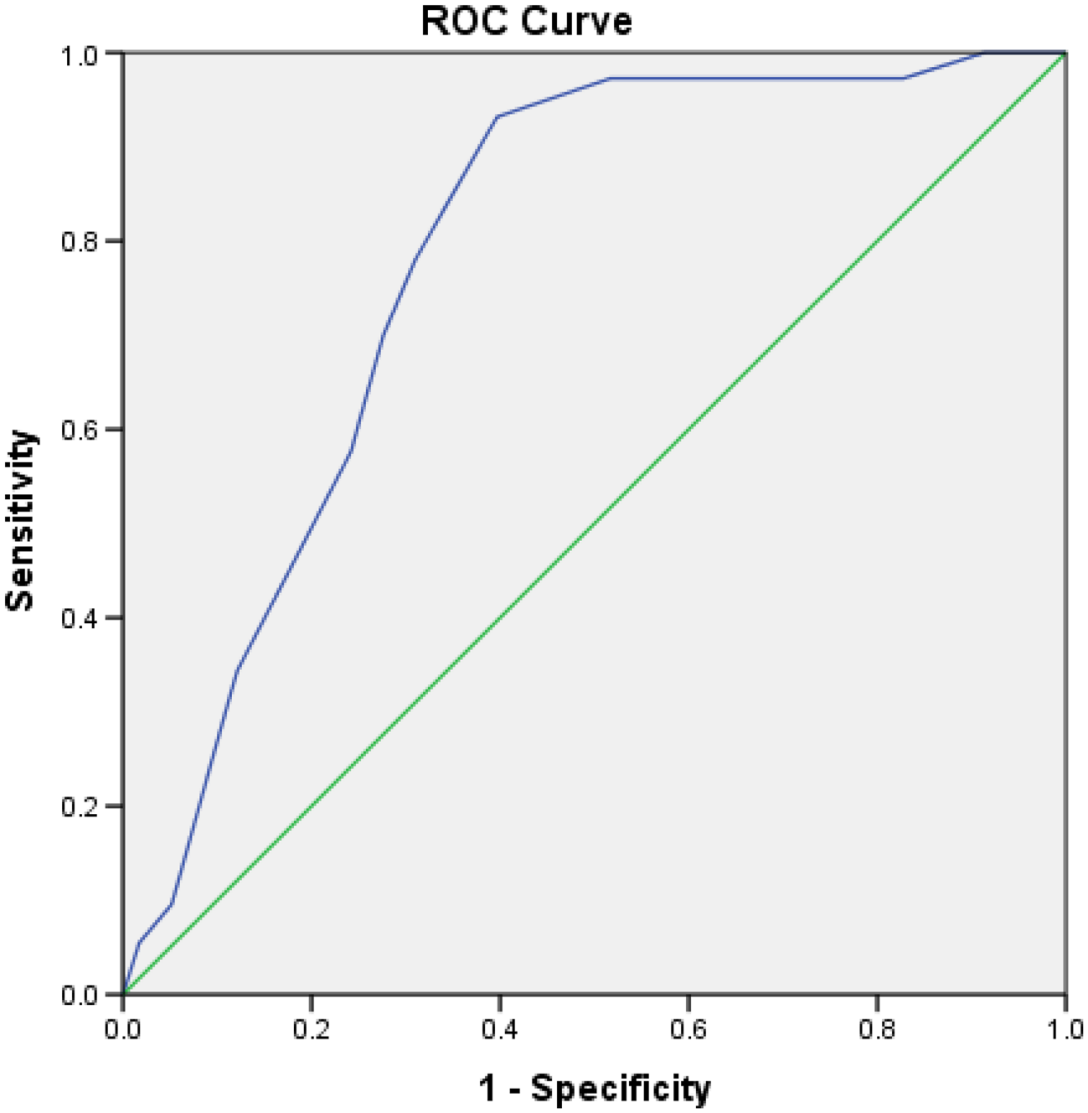
Receiver operating characteristic (ROC) curve for composite FMS score and MSI. The straight line showed the reference line, which was approximated by the ROC curve plotted on sensitivity (true positive rate) over 1-specificity (false positive rate) for each score total of the Functional Movement Screen (range, 0–21). Coordinates of the ROC curve showed that the composite FMS score corresponding best with the upper left hand portion of the curve was between 13.5 and 14.5 demonstrating the optimal cut-point score of 14 for the volleyball players participating in the study.

The ROC curve analyses (Fig. 1) for FMS composite scores of the volleyball players in this study determined a score of 14 as the most optimal cut-point. The coordinates of the curve indicated that the composite FMS score of 14 maximized both SN = 0.932, and SP = 0.603 values (Youden's index = 0.535). The score of 14 was then considered to be the proper cut-point for analysis.

Diagnostic and predictive values (e.g. sn, sp, PV+ and PV−, LR+ and LR−, ORs, and RR) of the optimal cut-point FMS composite score of 14 for the current study sample was then determined (Table 3).

**Table 3:** Diagnostic value of the FMS test for predicting musculoskeletal injuries in volleyball players.

Item	Value
Sensitivity	0.932
Specificity	0.603
Positive predictive value	0.747
Negative predictive value	0.875
Positive likelihood ratio	2.34
Negative likelihood ratio	0.112
Area under the curve	0.783
Odds ratio for (≤ 14/˃14)	0.048
Relative risk for being injured	3.46

Considering the predefined composite score of 14 as the cut-point (by previous studies^23,24^), the association between injury occurrence and FMS composite score was significant, χ^2^ (1, N = 131) = 43.60, *p* = 0.000. Those participants with FMS composite score of ≤ 14 were more likely than those with FMS composite score ˃14 to sustain injury. The association between injury occurrence and FMS composite score is reported in Table 4 and the odds ratios and the relative risk ratios are reported in Table 3. The odds ratio for (≤ 14/˃ 14) was 0.048. The relative risk for being injured was 3.46. Positive likelihood ratio was 2.34, and negative likelihood ratio was 0.11.

**Table 4:** Association between injury risk and FMS composite score.

Variable	Variable range	Injury	No injury	Total
FMS score (maximum = 21)	≤ 14	35	5	40
	> 14	23	68	91
	Total	58	73	131

## Discussion

Year-round participation in volleyball by approximately 200 million players all over the world predisposes a great number of volleyball players to inherent risk for sustaining injury. Unfortunately, athletes are often subjected to strain that can cause injury during training and competition; so, to assess movement patterns in daily sport practice, it is important to detect functional deficits. As a consequence, besides anthropometric characteristics, the identification of deficits in neuromuscular ability is another important parameter. The FMS is a useful screening tool to evaluate asymmetries, dysfunctions, and compensatory movement patterns in athletes^25^. The FMS has been developed to evaluate fundamental movements, which can suggest whether there is proper stability and mobility in athletes^25^. Identifying modifiable intrinsic risk factors related to volleyball injury can be highly valuable for volleyball players’ healthcare. The purpose of this study was to investigate the prognostic accuracy of the FMS in predicting youth volleyball injury through the national premier league of volleyball. The main finding of this study was that the FMS composite score recorded as a pre-season screening test could predict the probability of sustaining MSI through the competitive training time of a volleyball season.

Forty athletes scored at or below the predefined cut-point score of 14 and 35 out of the 40 sustained injuries. More comprehensive data are reported in Table 4.

As seen in Table 5, the mean FMS composite score for youth volleyball players in this study was 15.85 ± 3.31, which was similar to that reported by Mokha et al.^19^ for a different group of participants (NCAA Division II athletes, rowing, volleyball and soccer: 15.84 ± 1.73) but lower than that of Kiesel et al.^26^ for professional American football players (16.9 ± 3.0).

**Table 5:** Demographic characteristics and functional movement screen scores for men (n = 100) and women (n = 31).

Variable	Mean ± SD	Range
**Both men and women**		
Age (years), (N = 131)	15.87 ± 1.81	13–20
Height (cm), (N = 131)	176.30 ± 7.07	160–192
Weight (kg), (N = 131)	67.56 ± 6.18	56–82
Hand length (cm), (N = 131)	88.20 ± 4.84	72–100
Leg length (cm), (N = 131)	89.79 ± 4.87	75–102
Deep squat (N = 131)	2.46 ± 0.78	0–3
Hurdle step (N = 131)	2.39 ± 0.67	0–3
In-line lunge (N = 131)	2.15 ± 0.94	0–3
Shoulder mobility (N = 131)	2.35 ± 0.92	0–3
Active straight-leg raise (N = 131)	2.05 ± 0.74	1–3
Trunk stability push-up (N = 131)	2.48 ± 0.72	0–3
Rotatory stability (N = 131)	1.94 ± 0.74	0–3
FMS composite score (N = 131)	15.85 ± 3.31	6–21
**Men**		
Age (years), (N = 100)	16.51 ± 1.58*	14–20
Height (cm), (N = 100)	178.74 ± 5.71*	167–192
Weight (kg), (N = 100)	68.32 ± 6.42*	56–82
Hand length (cm), (N = 100)	89.60 ± 4.29*	79–100
Leg length (cm), (N = 100)	91.39 ± 3.29*	82–102
Deep squat (N = 100)	2.56 ± 0.72*	0–3
Hurdle step (N = 100)	2.39 ± 0.70	0–3
In-line lunge (N = 100)	1.98 ± 0.98*	0–3
Shoulder mobility (N = 100)	2.33 ± 0.93	0–3
Active straight-leg raise (N = 100)	1.94 ± 0.73*	1–3
Trunk stability push-up (N = 100)	2.60 ± 0.61*	1–3
Rotatory stability (N = 100)	1.86 ± 0.72*	0–3
FMS composite score (N = 100)	15.66 ± 3.36	6–21
**Women**		
Age (years), (N = 31)	13.83 ± 0.63	13–15
Height (cm), (N = 31)	168.45 ± 5.09	160–178
Weight (kg), (N = 31)	65.12 ± 4.63	58–75
Hand length (cm), (N = 31)	83.70 ± 3.68	72–89
Leg length (cm), (N = 31)	84.64 ± 3.29	75–90
Deep squat (N = 31)	2.16 ± 0.89	0–3
Hurdle step (N = 31)	2.41 ± 0.56	1–3
In-line lunge (N = 31)	2.70 ± 0.46	2–3
Shoulder mobility (N = 31)	2.41 ± 0.92	0–3
Active straight-leg raise (N = 31)	2.41 ± 0.67	1–3
Trunk stability push-up (N = 31)	2.12 ± 0.92	0–3
Rotatory stability (N = 31)	2.22 ± 0.76	1–3
FMS composite score (N = 31)	16.48 ± 3.11	10–21

*The mean differences are significant comparing to the female participants at the *p* < 0.05 level.

The ROC curve analysis demonstrated a cut-point score of 14 for the youth volleyball players participating in this study. The area under the curve was 0.783 (with sensitivity and specificity of 93% and 60%, respectively), demonstrating more than a 50% chance of predicting injury with the FMS composite score. These results contrast with those from Mokha et al.^19^ reporting an optimal cut-point score of 16, and an area under the curve as 0.363 (with sensitivity and specificity of 26% and 59%, respectively), which demonstrates a chance of less than 50% for predicting injury with the composite FMS score. However, present results are consistent with those from Kiesel et al. who reported an optimal cut-point score of 14 with sensitivity and specificity of 54% and 91%^23^. respectively, demonstrating a chance of more than 50% for predicting injury with the composite FMS score. O’Connor et al.^24^ in the first large scale study investigating the prognostic value of composite FMS scores in Marine officer candidates have also reported that FMS composite scores ≤ 14 were associated with increased injury risk^24^, which is consistent with the present study’s results as well.

Examining studies that contrast with the current results^19,22^ and those consistent with current results^23,24^, indicates that ongoing controversies exist. These differences could be due to the non-homogenized populations the researchers used for their investigations (e.g. various athletes from different types of sports^19,22^ as compared to investigating an individual group or type of athletes)^23,24^. Unlike Mokha et al.^19^ who did not report promising findings regarding predictive ability of composite FMS scores for MSI in NCAA Division II athletes (including various athletes from different types of sports), the current study’s results, similar to those of Kiesel et al.^23^, demonstrated that FMS composite scores can be useful in predicting MSI in a population of athletes participating in an individual sport such as youth volleyball as seen in the current study, and football in the study of Kiesel et al.^23^. O’Connor et al. investigating the Marine officer candidates also reported that FMS composite scores ≤ 14 are associated with increased injury risk^24^. This is also relatively consistent with the hypothesis that FMS composite scores of ≤ 14, if investigating a homogenized population or an individual group of people, can be useful in predicting MSI. Every individual type of sport and/or physical activity, due to its unique nature and requirements, leads to strengthening of individual muscles, and specificity of performance. Thus, it is likely that since the FMS includes some tests focused on the lower limbs, only one test focused on the upper limbs, and several that include function of both the upper or lower limbs and the core, a football player may potentially record a different FMS composite score than a basketball player. This supposition may have affected the outcomes of studies that include a non-homogenized population of athletes. A lower FMS composite score in two athletes of different sports could simply be attributed to the fact that a basketball player performs differently than the football player due to having different sporting requirements for strength, proprioception, balance, and mobility of lower limbs^27–30^. It cannot therefore be expected that recording a lower FMS composite score by an individual type of athlete necessarily makes them more susceptible to injury compared to another individual type of athlete. It is then suggested that in order to evaluate the predictive ability of the FMS composite score, previous researchers might have been better to investigate a homogenized population rather than different individual groups or types of athletes.

In light of the hypothesis mentioned in the previous paragraph, another interesting finding of this study was that female participants recorded better individual FMS scores than male participants in active straight-leg raise, rotatory stability, and in line lunge tests. The better scores of female participants in these three out of the seven individual FMS scores may be attributed to the better balance and muscular flexibility that has been described in females compared to males^31–33^. The male volleyball players in this study however, demonstrated better performance on push up- and deep squat- tests. The better results for these two individual tests in the male volleyball players may be attributed to greater strength generally demonstrated in males as compared to females^31,32^. Despite existence of significant differences in several FMS individual tests between the included male and female participants, the mean value of the FMS composite scores for the male and female participants were not significantly different. Regarding the comparison of FMS composite and individual scores between male and female volleyball players it should be noted that there was an almost 3-year age gap in the mean age between the male vs female volleyball players of this study that might have affected our findings. Chimera, Smith, and Warren noted that injury history and sex affect FMS and researchers should consider adjusting for confounders^34^. Since we did not consider these factors when designing this study, it is therefore suggested that a future study should investigate the predictive ability of FMS composite scores between two more age-homogenized and equivalent groups of male and female volleyball players separately, in order to compare these two groups and also in order to further estimate how much the FMS can identify the risk of sustaining injury in the two different sexes. This information may be able to explore the difference among predictive ability of FMS composite scores across male and female volleyball players related to measures of their differences in balance, flexibility, and strength.

The optimal cut-point score determined for the current study was exactly the same as the predefined cut-point of 14 by several other researchers^23,24,35^. Despite distinct differences in subjects and methodology (e.g. volleyball players, football players, Marine officer candidates), the findings of these studies were consistent, demonstrating that FMS composite scores ≤ 14 can identify some athletes with a greater chance of sustaining MSI over the course of the season. Therefore, this study highlights the effectiveness and usefulness of FMS as a valuable pre-seasonal screening test for youth volleyball athletes.

The result of a study related to FMS scores in different sports demonstrated that the rugby players showed a higher number of asymmetrical and dysfunctional movements than the other athletes (*p* < 0.01), while the highest scores in FMS components of shoulder mobility and active straight leg raise were obtained by the volleyball players (*p* < 0.01). In addition, most of the asymmetrical and painful movements in the athletes were measured on the shoulder mobility test^36^. Another study has also challenged the effects of sex and age: the results reported in this study suggest that age is a moderate determinant of FMS scores, whereas sex is not a determinant in this battery test in school age children (6–11 years old). Campa et al^36^ also highlighted that BMI is the primary predictor of FMS total score in school age children, but with a significant additional contribution from age, whereas the sex is excluded from this model. The current study has some limitations. Although all players were equivalent in their chosen sport and level of competition, an unequal distribution of female and male athletes were included which could have affected the findings of the study. It is therefore suggested to recruit more female volleyball players to address this limitation in future studies. Furthermore, the operational definition for MSI was having limitations in participating in at least one day of training or competition and since volleyball players already knew about the aim of the study, some athletes may have hidden some of their injuries from their team/club athletic trainers, thinking that this could have negatively affected their resume. It is therefore suggested that future investigators should be aware of this limitation and make efforts to blind the volleyball players to the injury recording phase of the study. Since we excluded players with previous injury from participating in this study, another limitation that is not addressed in this study is the influence of previous injury per se. it is therefore suggested that the future studies consider addressing how adjusting/accounting for previous injury may also influence injury risk and future studies may also look to see how this may affect FMS cutoff scores in specific athletic populations. Finally, because the sample came from a single country and level of participation, the findings of this study cannot be generalized to other levels of volleyball athletes around the world.

## Conclusion

The results of the current study indicate that the FMS composite score recorded during a pre-season screening test can predict the occurrence of MSI in youth volleyball players through a volleyball season. Specifically, a score of ≤ 14 was able to predict MSI with specificity of 0.60 and sensitivity of 0.93 in male and female youth premiere volleyball players. The odds ratio for (≤ 14/˃14) was 0.048. The relative risk for being injured was 3.46. Positive likelihood ratio was 2.34, and negative likelihood ratio was 0.11. Thus, it is suggested that volleyball players with a FMS composite score ≤ 14 should be advised to be assessed for the need for injury prevention strategies based on their individual FMS scores.

## Data Availability

The data that support the findings of this study are available from the corresponding author, MH, upon reasonable request.
